# A counterbalanced cross-over study of the effects of visual, auditory and no feedback on performance measures in a simulated cardiopulmonary resuscitation

**DOI:** 10.1186/1472-6955-10-15

**Published:** 2011-08-02

**Authors:** Carolyn L Cason, Cynthia Trowbridge, Susan M Baxley, Mark D Ricard

**Affiliations:** 1 College of Nursing, University of Texas at Arlington, Arlington, Texas, USA; 2 Department of Kinesiology, University of Texas at Arlington, Arlington, Texas, USA

## Abstract

**Background:**

Previous research has demonstrated that trained rescuers have difficulties achieving and maintaining the correct depth and rate of chest compressions during both in and out of hospital cardiopulmonary resuscitation (CPR). Feedback on rate and depth mitigate decline in performance quality but not completely with the residual performance decline attributed to rescuer fatigue. The purpose of this study was to examine the effects of feedback (none, auditory only and visual only) on the quality of CPR and rescuer fatigue.

**Methods:**

Fifteen female volunteers performed 10 minutes of 30:2 CPR in each of three feedback conditions: none, auditory only, and visual only. Visual feedback was displayed continuously in graphic form. Auditory feedback was error correcting and provided by a voice assisted CPR manikin. CPR quality measures were collected using SkillReporter^® ^software. Blood lactate (mmol/dl) and perceived exertion served as indices of fatigue. One-way and two way repeated measures analyses of variance were used with alpha set *a priori *at 0.05.

**Results:**

Visual feedback yielded a greater percentage of correct compressions (78.1 ± 8.2%) than did auditory (65.4 ± 7.6%) or no feedback (44.5 ± 8.1%). Compression rate with auditory feedback (87.9 ± 0.5 compressions per minute) was less than it was with both visual and no feedback (p < 0.05). CPR performed with no feedback (39.2 ± 0.5 mm) yielded a shallower average depth of compression and a lower percentage (55 ± 8.9%) of compressions within the accepted 38-50 mm range than did auditory or visual feedback (p < 0.05). The duty cycle for auditory feedback (39.4 ± 1.6%) was less than it was with no feedback (p < 0.05). Auditory feedback produced lower lactate concentrations than did visual feedback (p < 0.05) but there were no differences in perceived exertion.

**Conclusions:**

In this study feedback mitigated the negative effects of fatigue on CPR performance and visual feedback yielded better CPR performance than did no feedback or auditory feedback. The perfect confounding of sensory modality and periodicity of feedback (visual feedback provided continuously and auditory feedback provided to correct error) leaves unanswered the question of optimal form and timing of feedback.

## Background

Poor quality cardiopulmonary resuscitation (CPR) in in-hospital cardiac arrest among well-trained hospital staff [1] provided the impetus for the development and implementation of audiovisual feedback devices for use during clinical resuscitation. Audio and visual feedback improves the rate at which compressions are delivered so that they meet the rate recommended by the American Heart Association (AHA) Guidelines [2], but feedback does not consistently provide the same improvements in chest compression depth. Sugarman et al. [3], for example, report that in in-hospital resuscitations providing audiovisual feedback on compression rate and depth resulted in acceptably high rates throughout CPR but that compression depth declined within the first 90 seconds and decayed significantly between 90 seconds and 3 minutes. Sugarman et al. [3] attribute rescuers' inability to maintain adequate depth of compressions with feedback to rescuer fatigue and do not relate it to the type or duration of feedback provided.

Kramer-Johansen et al.'s [4] study of the use of real time automated feedback (visual waveforms and verbal messages) during out of hospital resuscitation suggest that rescuers' inability to maintain adequate depth of compression may arise not from rescuer fatigue but from complexity of the feedback provided. Kramer-Johansen et al. evaluated CPR quality prospectively with and without feedback. Feedback improved CPR quality; however, when the complexity of the feedback increased (feedback version 2), the percentage of compressions of adequate depth declined sharply. During the study, ambulance personnel were permitted to turn off auditory feedback (verbal messages and tonal prompts) and 18 percent did so but all retained the visual feedback. Kramer-Johansen et al. suggest further investigation to identify the form of optimal feedback (visual, tonal, voice prompts) and the ideal hierarchy and intensity of feedback.

Purpose of the Study

To examine the relative contribution of rescuer fatigue and type of feedback on quality of CPR, specifically rate and depth of chest compressions, this study compared the quality of CPR when rescuers performed 30:2 CPR [2] on a manikin while receiving visual only, auditory only and no feedback. The specific questions that guided the study included

1. Is there a difference in CPR quality (percent correction compressions, rate of compressions per minute, percent of compressions delivered at a depth of 38 mm or more, percent of compressions without full chest recoil, and duty cycle) associated with feedback? Is there a difference in CPR quality associated with type of feedback: Visual or Auditory?

2. What differences occur in indicators of fatigue (blood lactate levels and perceived exertion) and how are these associated with differences in CPR quality?

3. What differences are there in decay in chest compression rate and percent of compressions delivered at a depth of 38 mm or more when CPR is delivered with and without feedback?

We hypothesize that feedback will improve the quality of CPR; however, the quality of CPR will decline when the feedback is too complex or distracting.

## Methods

Subjects participating in Trowbridge's et al. [5] study of quality of CPR were invited to participate in the feedback study. In the Trowbridge et al. study subjects performed CPR for 10 minutes, performed both 30:2 and hands-only CPR and received feedback only during the first 30 compressions. Performance data obtained from subjects as they performed 30:2 CPR serve as the no feedback data for this study. Several months after having participated in the Trowbridge et al. study, subjects returned to participate in the feedback study. The study received approval from the University of Texas at Arlington institutional review board and is in compliance with the Helsinki Declaration (IRB No: 2009-1646).

### Design

The study design for collecting performance data on the effects of auditory only and visual only feedback was an experimental crossover design in which starting condition was randomly determined with the restriction that half of the subjects perform CPR while receiving auditory only feedback first and the other half receiving visual only feedback first. All subjects performed CPR under both conditions; feedback conditions were counterbalance.

Visual feedback consisted of a visual display of the depth of each compression. The display refreshed after every 20 compressions. When subjects performed CPR, they were directed to watch the display to judge and adjust compression rate and depth.

Auditory feedback was that which is routinely provided by the voice assisted manikin (VAM) ResusciAnne Skillreporter (Laerdal Medical Corporation, Stavanger, Norway). The voice-assisted manikin provides feedback to correct rate, depth, and pause between compressions and ventilations. Subjects were told to follow the instructions provided by the manikin as they performed CPR.

### Subjects

To be eligible for inclusion a subject had to have participated in the Trowbridge et al. [5] study, hold current certification in basic life support for healthcare professionals, and continue to be in good health (no prior or existing cardiopulmonary, musculoskeletal, or neuromuscular pathology). Twenty subjects participated in the Trowbridge et al. study. Two who participated in the Trowbridge et al. study were ineligible to participate in the feedback study for health reasons: one was pregnant and the other had a fractured extremity. Three were unable to schedule time for participation. Fifteen subjects were deemed to be enough for a power of 80 as the Cohen effect sizes reported by Trowbridge et al. [5] ranged between 0.9 and 1.9 for measures of compression rate and depth.

Subjects had a body mass index between 20 and 31 kilograms per meter squared. They ranged in height from 155 to 178 centimeters (M = 164; SD = 7) with mass between 48.1 and 85.9 kilograms (M = 70; SD = 12). They ranged in age from 23 to 60 (M = 40; SD = 15). No assessment of overall fitness was obtained. None had performed CPR in response to a cardiac arrest. None had completed refresher training since participating in the Trowbridge et al. study. Each received compensation ($100/hour) for study participation.

### Measures

CPR quality data were captured as each subject performed 30:2 CPR on a Resusci Anne Skillreporter (Laerdal Medical Corporation, Stavanger, Norway). A laptop computer connected to the Skillreporter continuously captured data using software provided by Laerdal Medical Corporation. The manikin was calibrated to provide data on compressions that ranged in depth from 1 to 55 mm with 34.6 kilograms of force required to compress the chest to at least 38 mm. Data collected included total number of compressions, number of correct compressions (compressions delivered at the correct rate, to the correct depth, with hands in the correct position and with full release of pressure at the end of the compression), number of compressions delivered to a depth of at least 38 mm, number of compressions ending without full release of pressure on the chest, and duty cycle (duration of compression to total cycle time).

Fatigue was assessed using both subjective (perceived exertion) and objective measures (blood lactate levels). Subjects rated perceived exertion using the Borg Rating of Perceived Exertion scale [6]. A YSI 1500 Sport Lactate Analyzer (YSI Incorporated, Yellow Springs, OH) provided blood lactate levels from blood samples obtained from subjects via finger prick.

### Procedure

Each subject was re-consented and completed a health questionnaire to confirm continuing eligibility for the study. Subject attire, location and placement of the manikin with respect to the subject and the procedure for performing CPR were standardized using those reported by Trowbridge et al. Subjects donned spandex sports apparel (shorts, tank top, and swim cap) and knelt on a mat placed on the floor next to the manikin to deliver 30:2 CPR. Each was directed to perform CPR until her rating of perceived exertion reached 17 or she was told to stop after 10 minutes.

In the auditory feedback condition, the subject heard the feedback provided by the manikin throughout the 10 minutes whenever CPR quality differed from that recommended by the AHA Guidelines [2]. The feedback was corrective; when the rescuer's rate of compression dropped below 100 per minute, she was told to 'press faster'. In the visual feedback condition, the subject received feedback by watching the graphic display on a computer screen placed on the floor. The graphic display provided feedback continuously throughout the 10 minutes of CPR.

Blood samples, obtained via finger prick, were taken before performing CPR, at the end of CPR and again at 5 minutes after the end of CPR. At 5 minutes and again at the end of CPR, the subject provided a rating of perceived exertion (Borg scale).

Data collection occurred in a university-based exercise science research laboratory. Participants were scheduled so that only one subject was tested at any given time and the second session was scheduled a minimum of 48 hours after the first.

### Statistical Analysis

NCSS 2001 (Salt Lake City, UT) was used to perform all statistical analyses including evaluating each variable for normality using Kolmogorov-Smirov and Shapiro-Wilk normality tests. One-way general linear model analyses of variance evaluated the feedback type effects (none, auditory, visual) on rate, depth, and CPR performance data (percent correct compressions, average rate of compressions per minute, average depth of compressions, percent of compressions delivered at a depth of 38 mm or more, percent of compressions without full release of press and duty cycle). A 3 × 3 (feedback type [none, auditory, and visual] × time [pre-CPR, post-CPR, and 5 minutes post-CPR]) within-within repeated measures analysis of variance evaluated the feedback effects and time effects on blood lactate levels. A 3 × 2 (feedback type [none, auditory, and visual] x time [after 5 minutes of CPR and after 10 minutes of CPR]) within-within repeated measures analysis of variance evaluated feedback effects and time effects on ratings of perceived exertion. All analyses of variance were interpreted using a step-down approach. When interactions were not significant, simple effects were evaluated using Tukey-Kramer's post hoc tests. For all tests, alpha was set at 0.05.

## Results

### Effect of feedback on CPR Quality

All subjects performed 10 minutes of CPR in each of the feedback conditions. The means and standard errors for each of the CPR quality measures are presented in Table 1. There were significant feedback condition main effects for percent correct compressions (F(2,42) = 4.5; p = 0.01), percent compressions delivered to depth of 38 mm or more (F(2,42) = 10.1; p < 0.001), percent of compressions without full release of pressure (F(2,42) = 13.4; p < 0.0001), and duty cycle (F(2,42) = 3.9; p = 0.02).

**Table 1:** CPR performance variables with and without feedback (Mean ± SEM [95% Confidence Interval])

Quality of CPR Indicator N = 15	No feedback	Auditory Feedback	Visual Feedback
Percent Correct Compressions [correct depth, rate, and release]	44.5 ± 8.1^a ^(27.1-61.8)	65.4 ± 7.6 (40.1-72.7)	78.1 ± 8.2 (60.4-95.7)
N who achieved 90% or better	2 (13%)	1 (6%)	9 (60%)
Average Rate of Compressions [number per minute]	96.5 ± 0.7 (95.2-97.8)	87.9 ± 0.5 ^b ^(86.9-88.9)	94.9 ± 0.7 (93.5-96.4)
N with rate between 90 and 120 ^c^	8 (53%)	8 (53%)	8 (53%)
Percent compressions with 38 or more mm depth	55.5 ± 8.9 ^d ^(36.4-74.7)	80.5 ± 6.0 (67.6-93.4)	95.4 ± 1.9 (91.3-99.5)
N who achieved 90% or better	3 (20%)	4 (27%)	13 (86%)
Average depth of compressions (mm)	39.2 ± 0.5 ^e ^(35.3-43.1)	41.4 ± 0.3 38.4-44.3)	42.2 ± 0.3 (38.8-45.5)
N who achieved 38-50 mm	9 (60%)	13 (86%)	13 (86%)
Percent compressions without full release of pressure	0.7 ± 0.3 (0.02-1.3)	23.4 ± 6.2^f ^(10.1-36.8)	0.8 ± 0.5 (0.44-2.0)
N with 5% or more of compressions without full release	0	11 (73%)	0
Average duty-cycle	46.1 ± 2.1 (41.5-50.7)	39.4 ± 1.6^g ^(35.9-42.9)	42.5 ± 1.1 (40.2-44.8)
N with range .30-.50	14 (93%)	13 (86%)	15(100%)

^a ^No feedback yielded lower percentage of correct compressions than did visual feedback (p = 0.01).

^b ^Auditory Feedback yielded slower rate than did no and visual feedback (p < 0.05).

^c ^Guidelines recommend 100 per minute [2]; we report range as no one subject delivered exactly 100 compressions per minute.

^d ^No feedback yielded lower percentage of adequate compressions (38-50 mm) than did auditory and visual feedback (p < 0.05).

^e^No feedback yielded shallower average depth of compressions than did auditory and visual feedback (p < 0.05).

^f ^Auditory feedback yielded a greater percentage of compressions that were not fully released than did no and visual feedback (p < 0.05).

^g ^Auditory feedback yielded a smaller duty cycle than did no feedback (p < .05).

Examination of the confidence intervals associated with the post hoc analyses (Table 1) reveal that visual feedback yielded better CPR performance when the quality indicators were the percentage of correct compressions, the percent of compressions delivered to a depth of 38 mm or more, and the average depth of compressions. In each instance feedback (both visual and auditory) improved performance but only with visual feedback was performance significantly improved over that observed with no feedback.

The post hoc analyses also reveal that auditory feedback yielded performance that was not as good as that obtained with either visual or no feedback on the quality indicators of average rate of compressions, the percent of compressions with full release and the average duty cycle (see Table 1).

### Effect of Feedback on Rate and Depth

**Figure 1:**
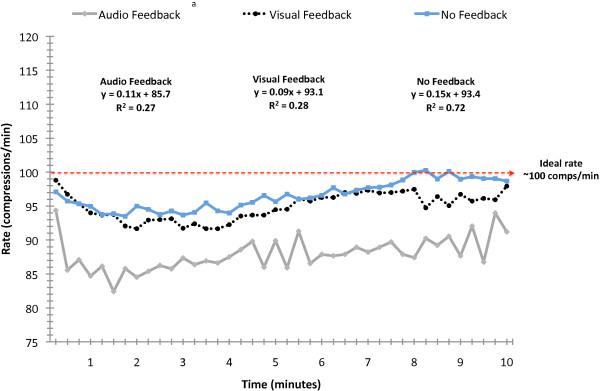
**Rate of CPR compressions over 10 minutes**. ^a^Main effect for condition (p < 0.001). Auditory feedback yielded significantly slower rate than did no feedback and visual feedback.

**Figure 2:**
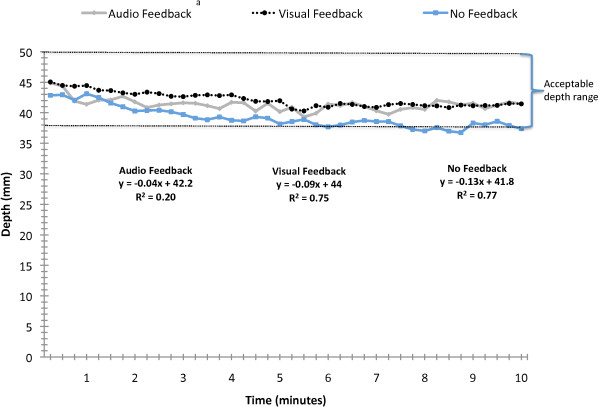
**Depth of CPR compressions over 10 minutes**. ^a^Main effect for condition (p < 0.001). No feedback yielded significantly less depth than did auditory and visual feedback.

Rate (compression per minute; Figure 1) and depth (mm; Figure 2) were analyzed using 15-second intervals. There were feedback condition main effects for rate (F(2, 1797) = 49.5; p < 0.0001) and depth (F(2, 1791) = 31.8; p < 0.0001). Post hoc analyses revealed that auditory feedback produced significantly slower compressions than did no feedback (p < 0.05) and that no feedback produced shallower compressions than did either auditory or visual feedback (p < 0.05).

**Figure 3:**
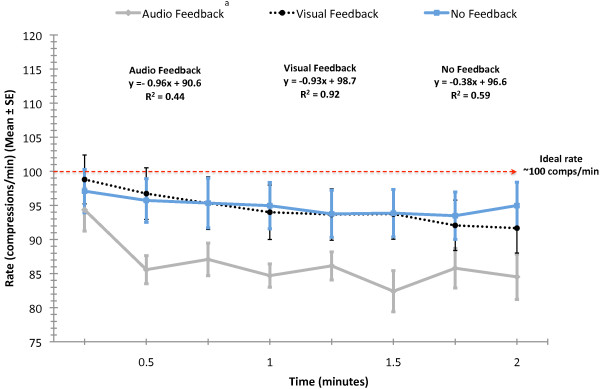
**Rate of CPR compressions over the first two minutes (Mean ± SE)**. ^a ^Main effect for condition (p < 0.0001). Auditory feedback yielded significantly lower rate than did no feedback and visual feedback.

**Figure 4:**
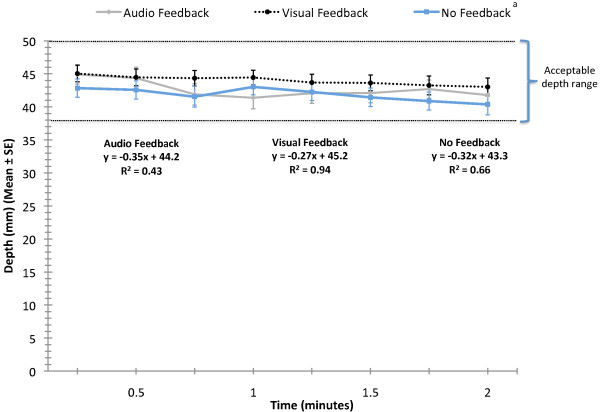
**Depth of CPR compressions over the first two minutes (Mean ± SE)**. ^a ^Main effect for condition (p = 0.008). No feedback yielded significantly shallower depth than did visual feedback.

Because the rate of decline in both rate and depth of compressions are considered vital components of successful CPR and the AHA Guidelines [2] recommend changing rescuers every two minutes, we analyzed these variables over the first two minutes of CPR. There were feedback condition main effects for rate (F(2, 14) = 17.2; p < 0.0001) and depth (F(2, 14) = 4.8; p = 0.008) in the first two minutes. Post hoc analyses revealed that auditory feedback yielded a significantly lower rate of compression than did either no or visual feedback (p < 0.05) (Figure 3). During the first two minutes, no feedback yielded significantly shallower depth than did visual feedback (p < 0.05), but depth in the no feedback condition was not different from depth in the auditory feedback condition (p > 0.05) (Figure 4). Linear trend lines for depth and rate over the first two minutes demonstrated negative slopes, indicating decreasing depth and rate of compressions. The R^2 ^values indicate that visual feedback yielded more consistent depth and rate over the first two minutes.

### Fatigue during CPR

**Figure 5:**
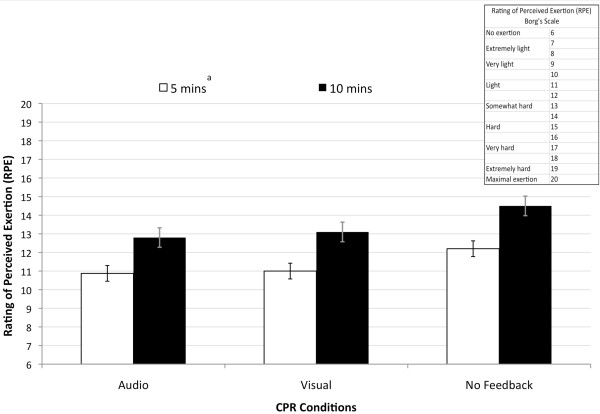
**Rating of Perceived Exertion during 10 minutes of CPR with and without feedback**. ^a ^Ratings of perceived exertion were less at 5 minutes than at 10 minutes (p < 0.05).

Ratings of perceived exertion and blood lactate levels (mmol/dL) were used to assess fatigue during the 10 minutes of CPR. There were no significant interactions; however, there were condition (feedback) main effects for ratings of perceived exertion (F(2, 28) = 8; p = 0.002) and lactate (F(2, 28) = 4.24; p = 0.04) and time main effects for ratings of perceived exertion (F(1,14) = 46.8; p < 0.001) and lactate (F(2,28) = 28.8; p < 0.001. Post hoc analyses revealed that in the no feedback condition subjects reported greater levels of exertion and, as expected, ratings of perceived exertion after 10 minutes of CPR were higher than at 5 minutes for all feedback conditions (Figure 5).

**Figure 6:**
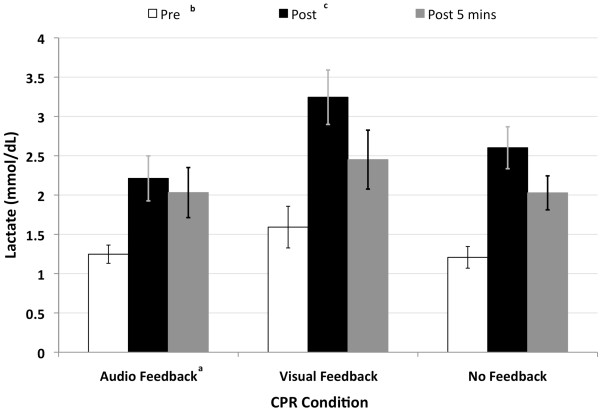
**Lactate concentration in blood during 10 minutes of CPR with and without feedback (Mean ± SE)**. ^a ^Audio feedback produced lower lactate concentrations than did either visual or no feedback (p < 0.05). ^b ^Pre-CPR lactate level were less than Post-CPR and Post 5 minutes CPR (p < 0.05). ^c ^Post-CPR lactate levels were greater than Post 5 minutes CPR (p < 0.05).

Blood lactate time effects indicate that lactate levels were higher at post and post 5 minutes than they were before CPR (Figure 6). Lactate levels remained elevated even after 5 minutes of rest and were significantly higher with visual feedback than they were with auditory feedback.

## Discussion

On each of the quality measures of CPR, the best average CPR performance occurred with visual feedback (on duty cycle visual feedback was better than auditory but equivalent to no feedback). A significantly larger percent of subjects achieved the CPR performance standards when doing CPR with visual feedback (no worse or better than no feedback on full release of pressure and duty cycle). Among these subjects, visual feedback also reduced performance variability; an effect observed in clinical resuscitation with audiovisual feedback [7]. Quality of CPR declined over the course of the 10 minutes but not significantly so when subjects received visual feedback.

Both auditory and visual feedback improved the percent of correct compressions (recommended rate, depth, and full release of pressure at the end of compression) and percent of compressions of adequate depth (38 mm or more) among females certified in basic life support performing 10 minutes of 30:2 CPR. Similar positive benefits of feedback in simulated resuscitation are reported by others using auditory feedback (VAM) [8-11] and audiovisual feedback (CPREzy™, Health Affairs, London, UK) [12,13].

Our results indicated that auditory feedback provided by the voice-assisted manikin (VAM) decreased the average rate of compressions, increased the percent of compressions without full release of pressure, and decreased duty cycle. Auditory feedback yielded performance that was significantly poorer than that observed with no feedback or with visual feedback. A potential hypothesis for this result is the nature of the feedback; it was error corrective occurring only when performance decayed to a preset threshold. Among the subjects in this study, average rate tended to drift downward with duration of CPR. When this occurred in the auditory feedback condition, the VAM advised the subject to 'press faster' and the subject responded by increasing the rate of compressions which gradually drifted back down to the threshold thereby increasing variability in compressions (displayed in Figures 1 and 3). One explanation for the observed performance variation is that the auditory advice disrupted the subject's CPR performance rhythm. Partial support for this hypothesis is provided in the results of Kern et al.[14] in simulated resuscitation and Chiang et al.[15] in clinical resuscitation. In each study, a metronome was used to provide continuous feedback during CPR and in each study compression rate improved significantly. Neither of these studies examined compressions without full release of pressure or duty cycle.

Incomplete release of force during decompression results in higher intra-thoracic pressures during the decompression phase of CPR which reduces venous blood flow to the heart and increases intracranial pressure which in turn decreases vital organ blood flow and the likelihood of survival [16-20]. Both Aufderheide et al. [19] and Niles et al. [20] report that feedback on release of pressure significantly decreases the number and percent of compressions with incomplete release of pressure during decompression. Aufderheide et al. [19] used an impedance threshold device (ResQPOD) to provide continuous feedback on intra-thoracic pressure during CPR while Niles et al.[20] used a commercial monitor/defibrillator system (Heartstart MRx Phillips, Andover, MA) to provide audiovisual directive and corrective feedback during clinical resuscitations. With corrective feedback Niles et al. report significant declines in the percent of compressions with incomplete release; but, that even with feedback, 28 percent of compressions had a residual force exceeding the threshold of 2.5 Kg estimated for use in pediatric CPR.

These results along with that reported by others [19,20] using error corrective feedback support Kramer-Johansen et al.'s [4] conclusions that complexity of feedback and its effects on performance need further examination. The results of this study and the residual error in CPR performance reported by others (e.g., Niles et al.[20]) suggest that there may be two different but equally important underlying constructs that need examination: sensory modality and periodicity of the feedback. These two aspects of feedback were completely confounded in this study as the visual feedback was continuous and the auditory feedback was periodic (error corrective). Kramer-Johansen et al.'s [4] results of audiovisual feedback during EMS clinical resuscitations in which personnel elected to turn off auditory feedback when given the option lends support to the notion that auditory feedback may be less useful than is visual feedback. On the other hand, the significantly better performance among subjects in this study when receiving visual feedback that was continuous as well as studies using metronomes [14,15] which provide continuous auditory feedback point to the superiority of continuous feedback over periodic feedback. Further study of single channel (visual or auditory) continuous and periodic feedback is needed to understand the unitary effects of both sensory modality and periodicity of feedback.

The results of this study argue against the hypothesis offered by Sugarman et al. [3] that decay in CPR quality even with feedback is due to rescuer fatigue. In this study, subject's ratings of perceived exertion were less with feedback than without it (Figure 5). Even though blood lactate levels (Figure 6) were significantly higher when these subjects performed 30:2 CPR with visual feedback their ratings of perceived exertion were not higher. In each feedback condition the blood lactate levels obtained before CPR were less than those at post and post 5 minutes CPR which indicated that the effort continued to be anaerobic. The elevated blood lactate levels with visual feedback coincide with the observation that subjects consistently had greater depth and rate when receiving visual feedback relative to receiving auditory feedback. This finding suggests that during visual feedback subjects were paying attention to their performance on the computer screen and possibly physiologically loading their anaerobic system to a greater level, but they did not experience a higher perceived fatigue.

Generalization of the results of this study to clinical resuscitation or to resuscitation training environments must proceed with caution as the study had several important limitations. The study was conducted in the laboratory where resuscitation was performed on a training manikin; one that did not simulate the changing force/pressure dynamics seen in human chests during resuscitation [21]. The study did not evaluate the kind of feedback most commonly seen in clinical resuscitation - a combination of auditory and visual feedback. However, the results of the study are sufficiently robust to warrant further study in simulated practice. Such studies should evaluate the comparative benefits of feedback when feedback (a) is continuous or real time versus error correcting or delayed and (b) uses single or multiple sensory modalities.

## Conclusions

In this study feedback mitigated the negative effects of fatigue on CPR performance and visual feedback yielded better CPR performance than no feedback or auditory feedback. The perfect confounding of sensory modality and periodicity of feedback (visual feedback provided continuously and auditory feedback provided to correct error) leaves unanswered the question of optimal form and timing of feedback.

## Competing interests

Carolyn L. Cason holds research grants from Laerdal Medical Corporation for other projects.

Cynthia Trowbridge declares no competing interests.

Susan M Baxley declares no competing interests.

Mark D. Ricard declares no competing interests.

## Authors' contributions

CLC conceived the study, participated in design and coordination, secured funding, assisted with data analyses, and helped draft the manuscript. CT carried out the study design, data collection, data analyses, and helped draft the manuscript. SMB assisted in data analyses and helped draft the manuscript. MDR carried out the study design, data collection, and data analyses. All authors read and approved the final manuscript.

## Authors' information

Carolyn L. Cason is Distinguished Professor and Associate Dean for Research. Her program of nursing and cardiopulmonary research spans over 30 years.

Susan M. Baxley recently completed her doctoral studies after over 30 years of experience in maternal health services. She teaches graduate level research courses.

Cynthia Trowbridge has been performing biomechanical and physiological research for over 10 years.

Mark Ricard has been performing biomechanical research for over 25 years.

## Pre-publication history

The pre-publication history for this paper can be accessed here:

http://www.biomedcentral.com/1472-6955/10/15/prepub
